# Genetic variants reshape the m^6^A epitranscriptome and drive transcriptomic reprogramming in colorectal cancer

**DOI:** 10.1038/s41598-025-23397-x

**Published:** 2025-11-11

**Authors:** Seung Hun Han, Seongmin Jang, Yeongwon Kim, Kun Tan, Miles F. Wilkinson, Hyobin Jeong, Junho Choe

**Affiliations:** 1 Department of Life Science, College of Natural Sciences, Hanyang University, Seoul, 04763 Republic of Korea; 2 Department of Obstetrics, Gynecology, and Reproductive Sciences, School of Medicine, University of California San Diego, La Jolla, San Diego, CA 92093 USA; 3 Institute of Genomic Medicine, University of California San Diego, La Jolla, San Diego, CA 92093 USA; 4 Department of Systems Biology, College of Life Science and Biotechnology, Yonsei University, Seoul, Republic of Korea; 5 Hanyang Institute of Bioscience and Biotechnology, Hanyang University, Seoul, 04763 Republic of Korea; 6 Research Institute for Natural Sciences, Hanyang University, Seoul, 04763 Republic of Korea; 7 Research Institute for Convergence of Basic Sciences, Hanyang University, Seoul, 04763 Republic of Korea

**Keywords:** N6‑methyladenosine, Genome-wide association study, Colorectal cancer, Alternative splicing, Epitranscriptomic regulation, Single-nucleotide polymorphisms, Transcriptomics, RNA, DNA, Bioinformatics, Gene expression analysis, Genomic analysis, Sequencing, Cancer genomics, Data integration, Genome informatics, Cancer genomics, Epigenomics, Eukaryote, Gene expression, Gene regulation, Mutation, Sequencing

## Abstract

Genome-wide association studies (GWAS) have identified numerous single-nucleotide polymorphisms (SNPs) associated with various diseases, including cancer. However, the mechanisms by which these SNPs contribute to disease susceptibility remain largely unclear. While recent studies have explored the transcriptional impact of disease-associated SNPs, their role in post-transcriptional regulation has been less extensively investigated. In this study, we investigated whether cancer-associated SNPs influence gene expression by altering *N6*-methyladenosine (m^6^A) RNA methylation. We collected GWAS-identified SNPs across nine cancer types and integrated these with matched tumor and normal m^6^A RNA immunoprecipitation sequencing (m^6^A-seq) and RNA sequencing (RNA-seq) datasets. We first identified differentially methylated m^6^A sites and assessed whether cancer-associated SNPs were enriched within these regions. These analyses revealed that cancer-associated SNPs were significantly enriched within hypermethylated m^6^A regions in colon cancer. Integrative analysis revealed that SNPs enriched in m^6^A-modified regions are associated with altered gene expression and RNA splicing, suggesting that m^6^A methylation mediates the post-transcriptional impact of genetic variants. Experimental validation further confirmed altered gene expression following ALKBH5 knockdown, consistent with patient-derived data. Collectively, our findings support a novel mechanistic connection between genetic variants and RNA methylation-driven transcriptomic regulation in colorectal cancer, underscoring the epitranscriptome as a potential axis of oncogenic control.

**Supplementary Information:**

The online version contains supplementary material available at 10.1038/s41598-025-23397-x.

## Introduction

*N6*-methyladenosine (m^6^A), the most abundant internal modification in eukaryotic messenger RNAs (mRNA)^1,2^, plays a pivotal role in shaping the fate of transcripts by influencing RNA stability^3,4^, splicing^5,6^, localization^5,7^, and translation^4,8,9^. The deposition and removal of m^6^A are orchestrated by methyltransferase (“writer”) and demethylase (“eraser”) enzymes, whereas RNA-binding proteins (“readers”) specifically recognize the modification and mediate downstream regulatory effects on RNA metabolism, establishing a dynamic and reversible control layer^10,11^. Accumulating evidence suggests that dysregulation of m^6^A contributes to a wide range of human diseases, including cancer^12–14^, where it contributes to tumor initiation, progression, and immune evasion by reshaping transcriptomic outputs in both oncogenes and tumor suppressors^11,14–16^.

Despite increasing interest in m^6^A-dependent gene regulation, the extent to which genetic variants contribute to shaping the m^6^A landscape remains poorly defined, although epigenetic and environmental factors have been shown to influence its dynamics^17,18^. Given that m^6^A deposition depends on specific sequence and structural contexts such as the DRACH motif (D = A/G/U, R = A/G, H = A/C/U)^19^, it is plausible that single-nucleotide polymorphisms (SNPs) may disrupt or create m^6^A consensus sites^20^, thus altering the modification status of target transcripts in a genotype-dependent manner^21^. Such changes could in turn affect RNA stability or processing, ultimately influencing post-transcriptional gene regulation^22,23^.

Genome-wide association studies (GWAS) have uncovered numerous SNPs associated with cancer susceptibility^24,25^. However, the majority of these SNPs are located in noncoding regions of the genome^26^, making it difficult to assign their biological functions. Traditional functional interpretations have focused on the potential for noncoding SNPs to affect transcription factor binding, enhancer activity, promoter accessibility, or chromatin organization^27,28^. Although these models are well established, the potential of noncoding SNPs to act through post-transcriptional mechanisms remains underexplored. Recent work has suggested that RNA modifications, particularly m^6^A, may constitute an emerging layer of gene regulation through which genetic variants influence the transcriptome^29–31^. Although some studies have begun to explore these possibilities, the functional consequences of SNP-driven m^6^A modulation remain largely uncharacterized. Nevertheless, comprehensive investigations into the mechanistic interplay between cancer-associated SNPs and the m^6^A methylome are still lacking and are needed to define their functional impact on the transcriptome.

In this study, we hypothesize that cancer-associated SNPs may contribute to transcriptomic changes by altering m^6^A RNA modification sites. To test this hypothesis, we performed an integrative analysis of GWAS data from nine cancer types, alongside matched m^6^A RNA immunoprecipitation sequencing (m^6^A-seq) and RNA sequencing (RNA-seq) datasets derived from tumor and normal tissues. We examined whether cancer-associated SNPs are enriched in regions with differential m^6^A methylation, and whether these variants are associated with changes in transcript levels or alternative splicing patterns. Our results reveal that m^6^A may function as a post-transcriptional mediator of genetic variant effects in cancer.

## Results

### Transcriptome-wide m^6^A profiling reveals distinct methylation landscapes between tumor and normal tissues

**Fig. 1:**
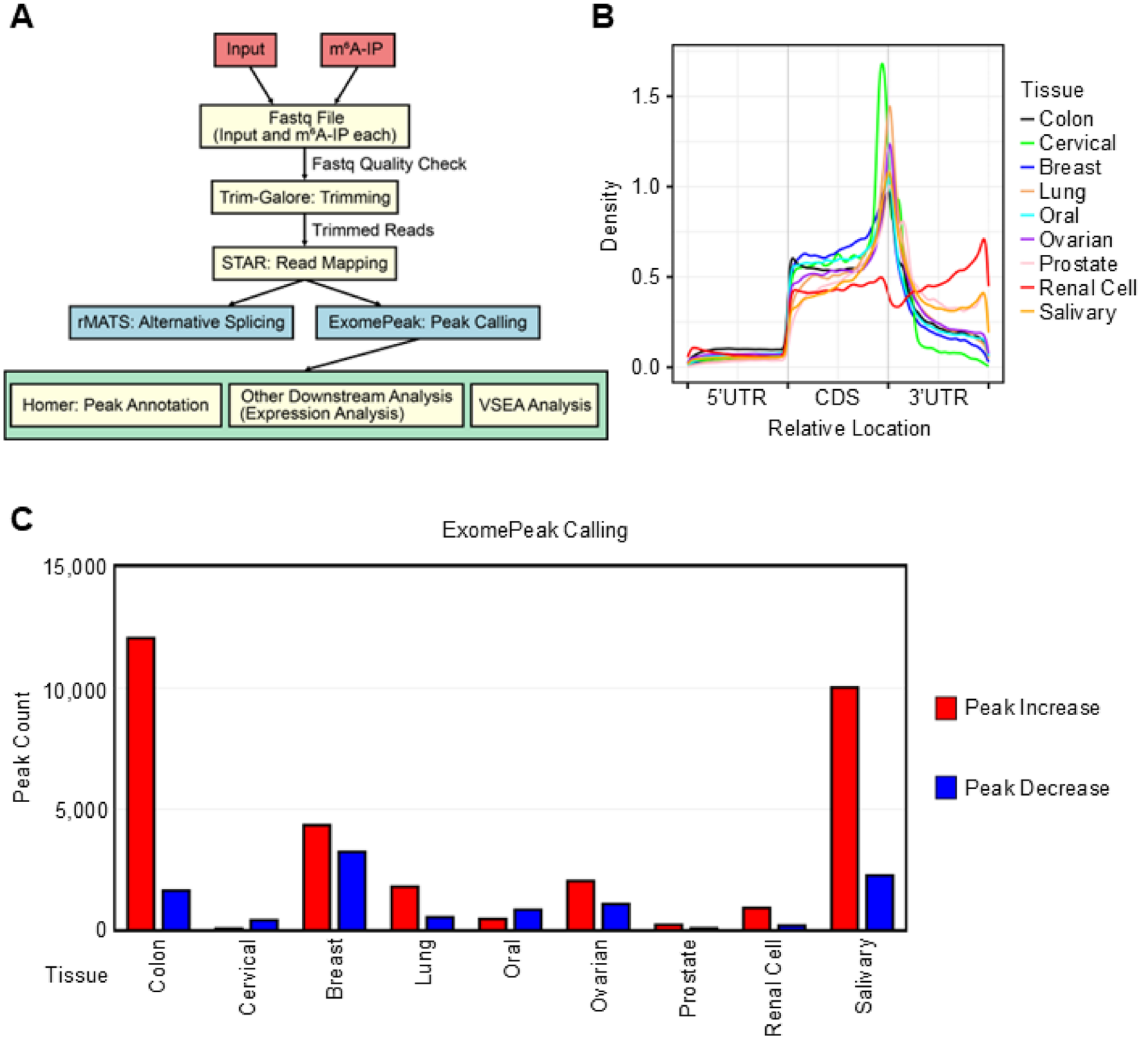
Overview of m^6^A-seq analysis pipeline and summary of peak distribution across various cancer types. (**A**) Schematic workflow of the analysis pipeline used in this study. (**B**) Metagene plot showing the distribution of m^6^A peaks along transcripts from various tissue types. (**C**) Bar plot of differential m^6^A peaks identified by exomePeak, showing the number of peaks increased (red) or decreased (blue) in tumor tissues across different cancer types (adjusted *P* < 0.05, after Benjamini-Hochberg multiple correction).

To examine the relationship between cancer-associated SNPs and alterations in m^6^A methylation patterns, we first analyzed m^6^A-seq data from nine cancer types and matched normal tissues. The m^6^A-seq datasets were obtained from the Sequence Read Archive (SRA) database (Supplementary Fig. S1A)^32^. Raw sequencing reads were preprocessed using Trim-Galore to remove adapters and low-quality bases, and aligned to the human reference genome (GENCODE hg38) using STAR^33^. The aligned data were subsequently used for gene expression quantification, alternative splicing detection, and m^6^A peak identification as summarized in the overall analysis pipeline (Fig. 1A). Gene expression levels in tumor and normal samples were quantified using HTSeq^34^, followed by differential expression analysis with DESeq2 to identify differentially expressed genes (DEGs) in tumors^35^. Alternative splicing differences were detected using rMATS-turbo to identify differentially spliced genes between tumor and normal tissues^36^.

We next identified m^6^A-enriched peaks from the m^6^A-seq data using exomePeak and annotated their locations within transcript features^37^. Motif enrichment analysis confirmed that these peaks were significantly enriched for the canonical DRACH sequence, a known motif for m^6^A deposition (Supplementary Fig. S1B)^38,39^. We also annotated the genomic distribution of all detected m^6^A peaks, including intronic, exonic, intergenic, and regulatory regions, to provide a more complete reference for downstream analyses (Supplementary Fig. S1C). Differential m^6^A peaks (DMPs) were identified by comparing m^6^A enrichment levels between tumor and normal samples. These DMPs served as the basis for subsequent analyses, including the evaluation of their overlap with cancer-associated SNPs and transcriptomic features in multiple cancer types.

To further characterize the biological relevance of m^6^A dynamics, we examined both the positional distribution and cancer-specific variation of m^6^A methylation. Using metaplotR, we visualized the density of m^6^A peaks across transcript regions and found that peaks were predominantly enriched in the 3′ untranslated regions (3′UTRs) and coding sequences (CDSs) across the nine cancer types analyzed (Fig. 1B)^40^. This distribution pattern is consistent with prior findings on the positional preference of m^6^A across transcripts^41^. In addition to these spatial patterns, previous studies have also reported that m^6^A levels vary across cancer types, with some cancers exhibiting increased m^6^A methylation while others display decreased levels compared to normal tissues^11,42,43^. In line with these findings, m^6^A modification changes exhibited substantial heterogeneity across cancer types, with both up-regulated and down-regulated DMPs identified (Fig. 1C). In particular, cancers exhibited increased m^6^A methylation in colon, breast, lung, ovarian, prostate, renal cell, and salivary cancers, with colon and salivary cancers showing a greater number of up-regulated DMPs compared to down-regulated DMPs. These results suggest that cancer-type-specific reprogramming of m^6^A methylation may contribute to transcriptomic remodeling in the tumor microenvironment, a possibility we explore further in the following analyses.

### Colon cancer-specific enrichment of genetic variants in differential m^6^A regions

**Fig. 2:**
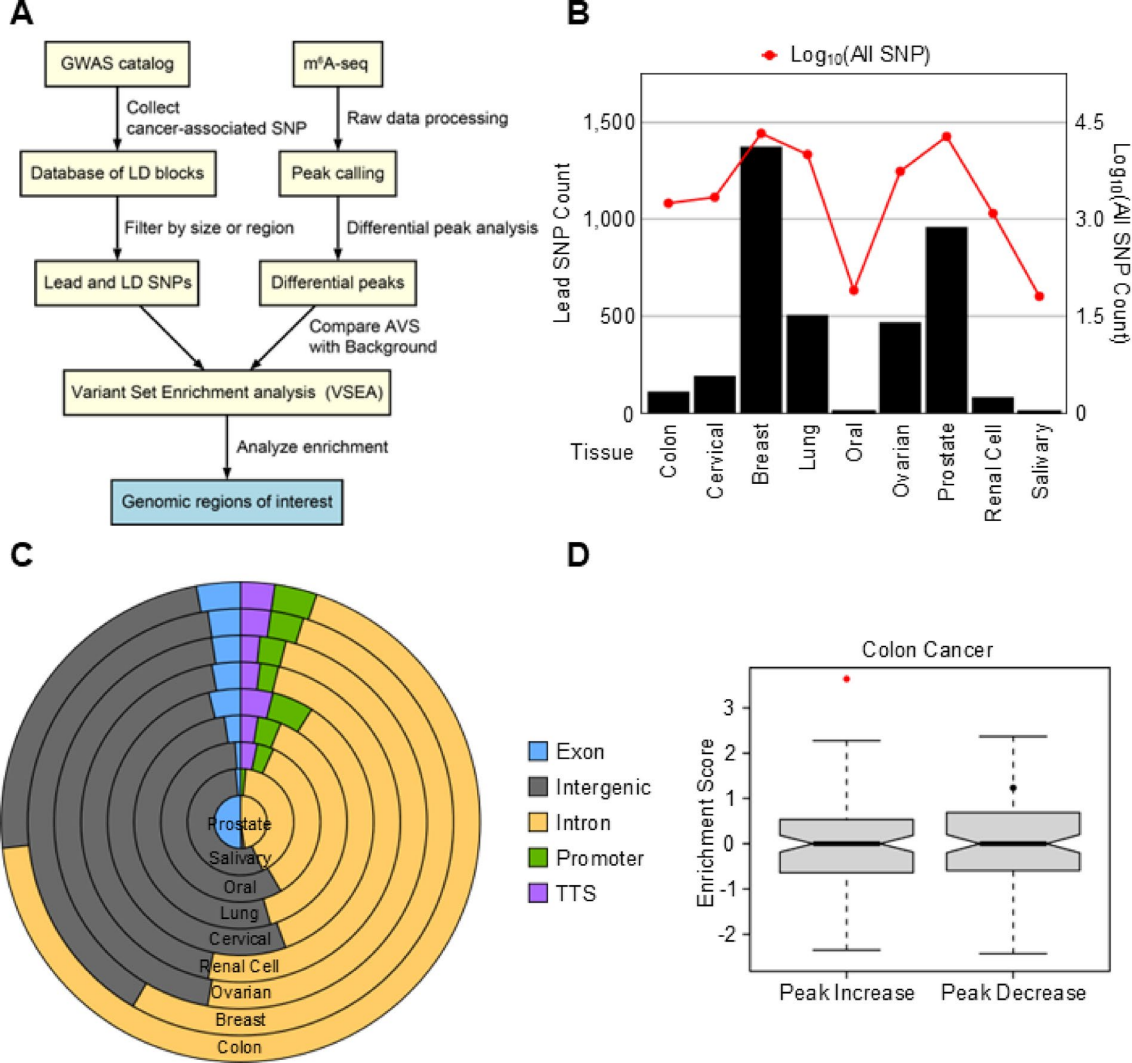
Integration of differential m^6^A peaks in multiple tumor tissues with cancer-associated SNPs via Variant Set Enrichment Analysis (VSEA). (**A**) Workflow of VSEA combining cancer associated SNPs from GWAS and m^6^A-seq data from different cancer types. (**B**) Bar plot shows the counts of lead SNP and the line plot shows the number of total SNPs (lead SNPs and LD SNPs) across cancer types. (**C**) Stacked pie graph shows the genomic distribution of cancer-associated lead SNPs in different cancer types. (**D**) Enrichment scores of colon cancer-associated SNPs within the differential m^6^A peaks based on VSEA. Enrichment scores were calculated separately for increased and decreased peaks in tumors. A red dot indicates statistical significance (*P* < 0.05, permutation test, *n* = 1,000).

To evaluate whether m^6^A changes were associated with genetic variation, Variant Set Enrichment Analysis (VSEA) was used to test for enrichment of cancer-associated SNPs within DMP regions (Figs. 1A and 2A)^44^. In this analysis, we integrated GWAS data with m^6^A-seq profiles across nine cancer types. We retrieved lead SNPs and their linkage disequilibrium (LD) SNPs from the GWAS Catalog to construct a comprehensive set of cancer-associated variants^45^. These SNPs were intersected with DMPs previously identified between tumor and normal samples (Fig. 1C). To assess whether this overlap was greater than expected by chance, we applied VSEA, comparing observed intersections against a randomized background (Fig. 2A). This analysis was designed to determine whether cancer-associated SNPs are non-randomly distributed within regions of altered m^6^A methylation, providing a potential mechanistic link between genetic variation and m^6^A epitranscriptomic regulation.

We first quantified the number of lead SNPs and total SNPs (lead + LD) associated with each cancer type to estimate the differences in genetic variant burden in different cancer types. As shown in Fig. 2B, breast and prostate cancers exhibited the highest number of lead SNPs, while colon, cervical, oral, renal cell, and salivary cancers displayed substantially fewer. This disparity implies that the contribution of inherited genetic variants to m^6^A regulatory dynamics may differ across tissue types.

To understand the genomic distribution of these SNPs, we annotated them by region, including exonic, intronic, intergenic, promoter, and transcription termination site (TTS) regions (Fig. 2C). As expected, most SNPs were located in intronic and intergenic regions, followed by exonic and regulatory elements, consistent with prior reports that disease-associated variants often reside in noncoding regions^46,47^.

We next examined whether DMPs, classified as increased peaks or decreased peaks in tumors compared to normal, were significantly enriched for cancer-associated SNPs in each corresponding cancer type. Strikingly, among all nine cancer types analyzed, only colon cancer showed a significant enrichment of cancer-associated SNPs within increased peaks (Fig. 2D; Supplementary Table S1; adjusted *P* = 0.0023 based on VSEA after Benjamini-Hochberg multiple correction). This enrichment was assessed using permutation-based sampling, comparing the observed overlap between SNPs and DMPs to a null distribution generated from randomly selected genomic regions. This finding indicates that genetic variation may play a unique role in shaping m^6^A remodeling in colon cancer, highlighting a genetic-epitranscriptomic axis that is specifically observed across certain tumor types.

### Colon cancer-associated SNPs overlapping with m^6^A methylation may contribute to transcriptomic changes

**Fig. 3:**
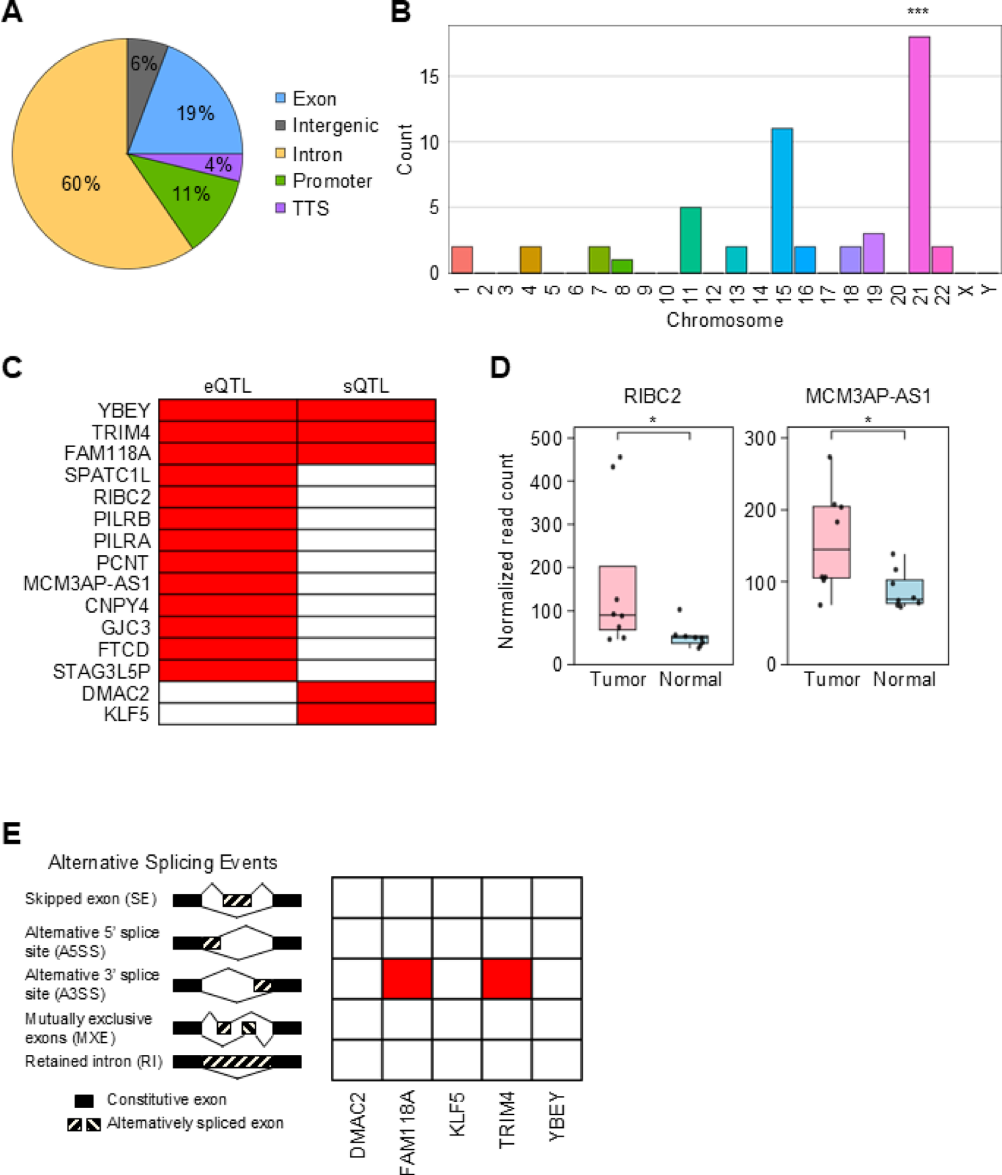
Functional impact of m ^6^ A-associated SNPs in colon cancer. (**A**) Genomic distribution of colon cancer-associated SNPs overlapping with increased m^6^A peaks. We define these SNPs as m^6^A-associated SNPs in colon cancer. (**B**) Chromosomal distribution of m^6^A-associated SNPs. (**C**) Annotation of m^6^A-associated SNPs using eQTLs and sQTL information based on snpXplorer^48^. Red box indicates the presence of eQTLs and/or sQTLs of corresponding SNPs. (**D**) Expression of *RIBC2* and *MCM3AP-AS1* in tumor vs. normal tissues based on RNA-seq data. Statistical significance was determined based on adjusted *P*-values (Wald test in DESeq2 followed by Benjamini-Hochberg multiple correction). (**E**) Evaluation of alternative splicing events for five sQTL-related genes detected using rMATS-turbo. Red indicates the presence of alternative splicing events of corresponding genes in colon cancers compared to normal. The asterisks indicate statistical significance: * *P* < 0.05, ** *P* < 0.01, *** *P* < 0.001.

Given that colon cancer exhibited significant enrichment of cancer-associated SNPs in their differentially m^6^A-modified regions, we next investigated whether these SNPs are directly positioned within altered m^6^A peaks. We performed an overlap analysis between colon cancer-associated SNPs and hypermethylated m^6^A peaks, followed by annotation of their genomic positions (Fig. 3A). Genomic annotation revealed that a majority of SNPs overlapping with hypermethylated m^6^A peaks in colon cancer were located in intronic regions (60%). This annotation summarizes the positional distribution of m^6^A-overlapping colon cancer-associated SNPs. A permutation test confirmed that the observed proportion of intronic SNPs was within the expected range given the genomic distribution of colon cancer-associated SNPs (adjusted *P* = 0.99986), suggesting no statistical enrichment. By contrast, SNPs located in exonic and promoter regions were statistically enriched compared to the null distribution (adjusted *P* = 0.000025 and 0.001625, respectively), indicating potential regulatory relevance. To facilitate further investigation, we compiled a comprehensive list of colon cancer-associated SNPs overlapping with hypermethylated m^6^A peaks, including rsIDs, genomic locations, eQTL/sQTL annotations, and m^6^A peak information (Supplementary Table S2). These overlapping SNPs were unevenly distributed across chromosomes, with higher numbers observed on chromosomes 11, 15, and 21 (Fig. 3B). To determine whether this chromosomal distribution reflected meaningful enrichment rather than random variation, we conducted a permutation test. In each of 100,000 iterations, we randomly sampled the same number of SNPs from the full set of colon cancer-associated SNPs and recorded their chromosomal locations. This generated a background distribution for SNP counts per chromosome. Compared to this background, chromosome 21 was significantly enriched (adjusted *P* < 0.05), while chromosomes 11 and 15 did not show enrichment beyond expectation. Previous cancer genomics studies have reported the recurrent chromosome arm level genetic rearrangement in chromosome 21q in microsatellite instability-positive (MSI) primary colon cancers, which aligns with our enrichment findings^48^. These findings suggest that chromosome 21 may harbor a higher density of functionally relevant cancer-associated SNPs within hypermethylated m^6^A regions.

To gain functional insight into the 52 colon cancer-associated SNPs located within DMPs and narrow down to the functionally associated SNPs, we annotated them as expression quantitative loci (eQTLs) or splicing quantitative trait loci (sQTLs) using the snpXplorer platform^49^. This analysis identified 25 SNPs located within hypermethylated m^6^A peaks in tumors, which were predicted to influence transcript expression and/or splicing (Supplementary Fig. S3A and Table S2). We subsequently extracted the target genes of these SNPs and categorized them according to QTL type to identify candidates potentially regulated at the level of expression, splicing, or both (Fig. 3C).

To assess whether these QTL-associated genes display differential transcript levels in tumors, we analyzed input RNA-seq data matched to the m^6^A-seq samples from colon cancer and corresponding normal tissues, as illustrated in the workflow in Fig. 1A. First, we identified DEGs in colon cancer compared to normal tissues and generated a heatmap representation of global expression changes (Supplementary Fig. S3B). By integrating these expression data with our eQTL results, we identified *RIBC2* and *MCM3AP-AS1* as genes that not only carried m^6^A-associated eQTL signals but were also significantly upregulated in colon tumors compared to normal tissues (Fig. 3D). These observations were further supported by TCGA pan-cancer data showing elevated expression of *RIBC2* mRNA and *MCM3AP-AS1* lncRNA in colon adenocarcinoma compared to normal tissues (Supplementary Fig. S3C and S3D). These findings support a possible mechanistic link between m^6^A-associated SNPs and altered gene expression in colon cancer. According to the TCGA pan-cancer data, both genes are also upregulated in multiple other cancer types compared to normal. This broader expression pattern is consistent with previous studies reporting that *RIBC2* and *MCM3AP-AS1* are differentially expressed and clinically relevant in various human cancers beyond colon cancer, likely through mechanisms independent of m^6^A regulation^50–52^. While these findings highlight the potential importance of these genes across multiple cancer types, the specific contribution of m^6^A-associated SNPs to their regulation remains to be elucidated. We compiled an extended list of colon cancer-associated m^6^A SNPs located within the *RIBC2* and *MCM3AP-AS1* loci, identified through integrative annotation including HaploReg (Supplementary Table S3). As a next step, we examined the potential involvement of m^6^A-associated SNPs in alternative splicing regulation in colon cancer. Using the same RNA-seq dataset, we performed a genome-wide splicing analysis to identify differentially spliced genes (DSGs) in colon cancer relative to normal tissue. This analysis revealed widespread splicing alterations in skipped exons (SE), alternative 5’ or 3’ splice sites (A5SS, A3SS), mutually exclusive exons (MXE), and retained introns (RI) (Supplementary Fig. S3E). Among the genes containing m^6^A-SNP overlaps, *FAM118A* and *TRIM4* showed significant A3SS alterations (Fig. 3E). These findings suggest that m^6^A-associated SNPs may contribute to alternative splicing regulation in a transcript-specific manner, potentially influencing cancer-associated splicing patterns.

### Genetic variants modulate m^6^A modification and drive post-transcriptional rewiring in colorectal cancer

**Fig. 4:**
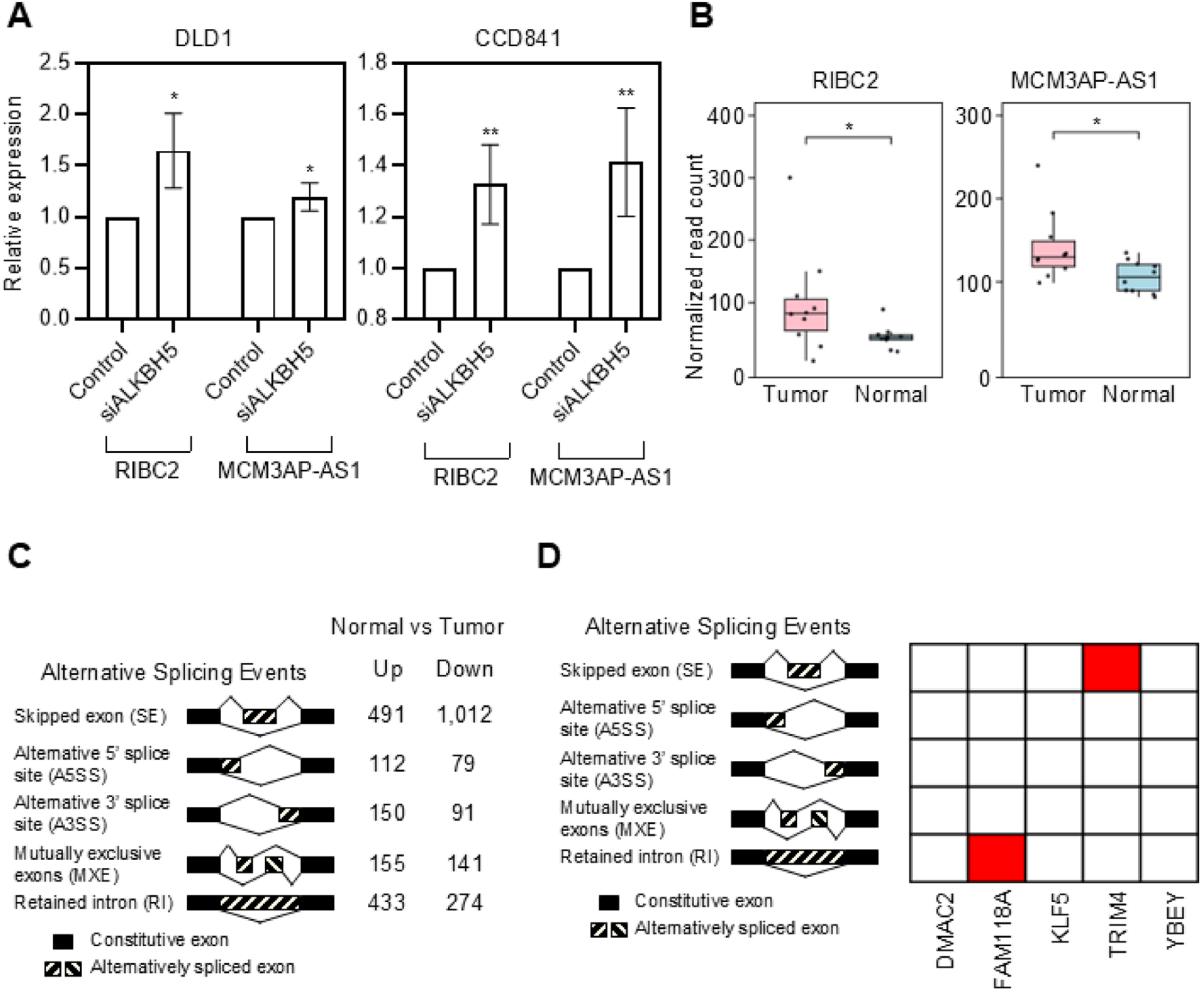
Orthogonal validation of differentially expressed genes and differential splicing events in colon cancer potentially linked with m^6^A-associated SNPs. (**A**) RT-qPCR showing increased expression of *RIBC2* and *MCM3AP-AS1* after ALKBH5 knockdown in DLD1 and CCD841 cells. Statistical significance was determined using two-tailed unpaired t-test. Error bars represent standard deviation (SD). (**B**) Upregulation of *RIBC2* and *MCM3AP-AS1* in colon tumor tissues compared to normal tissues based on orthogonal RNA-seq dataset (PRJNA742008). Statistical significance was determined based on adjusted *P*-values (Wald test in DESeq2 followed by Benjamini-Hochberg correction). (**C**) Global identification of differential splicing events between tumor and normal tissues categorized by event type. (**D**) Alternative splicing events of selected genes identified from the PRJNA742008 dataset using rMATS-turbo. Red boxes indicate the presence of alternative splicing events of corresponding genes in colon cancers compared to normal. The asterisks indicate statistical significance: * *P* < 0.05, ** *P* < 0.01.

The observed association between colon cancer-associated SNPs and altered m^6^A methylation, along with accompanying changes in gene expression and splicing, led us to investigate whether these transcriptomic alterations are mediated by m^6^A modification. We performed functional assays using the colon cancer cell line DLD1 and the normal colon epithelial cell line CCD841. In both cell lines, we inhibited m^6^A demethylation by knocking down ALKBH5, an m^6^A demethylase known to exert tumor-suppressive functions in colon cancer. In parallel, to assess concordance with patient-derived data and clinical relevance, we analyzed a publicly available dataset (PRJNA742008) containing matched m^6^A-seq and RNA-seq data from ten colon cancer patients. This complementary analysis allowed us to assess the regulatory effects of m^6^A-SNPs in both experimental systems and patient-derived tissues. Upon ALKBH5 depletion, both *RIBC2* and *MCM3AP-AS1* transcripts were upregulated, supporting the hypothesis that m^6^A modification promotes the expression of these genes. (Fig. 4A). Consistent with this observation, analysis of dataset from an orthogonal cohort of colon cancer patients (PRJNA742008) revealed that both genes were significantly upregulated in tumor tissues, supporting the reproducibility of our findings in an independent dataset (Fig. 4B).

Using transcriptome data from PRJNA742008, we additionally investigated differential splicing events potentially regulated by m^6^A-SNPs in colon cancer. Analysis with rMATS identified differential splicing events between tumor and normal tissues (Fig. 4C). Notably, *FAM118A* showed RI events, and *TRIM4* showed SE events in tumor tissues (Fig. 4D). These splicing alterations were distinct from the A3SS events observed in our earlier analysis, suggesting that m^6^A-associated SNPs may regulate splicing in a context- and transcript- dependent manner. Moreover, while earlier analyses emphasized increased A3SS in tumor tissues, our current findings reveal a broader spectrum of splicing changes, underscoring the complex regulatory roles of m^6^A-SNP interactions in colorectal cancer.

## Discussion

Our findings uncover a previously underappreciated regulatory axis suggesting that single-nucleotide polymorphisms (SNPs) may modulate the transcriptome through m^6^A RNA methylation. By integrating m^6^A-seq, RNA-seq, and genome-wide association studies (GWAS) across nine cancer types, we identified significant enrichment of cancer-associated SNPs within dynamically remodeled m^6^A regions.

Notably, among the nine cancer types analyzed, only colon cancer exhibited significant enrichment of SNPs within m^6^A peaks that gained methylation in tumor tissues, passing multiple testing correction. While we cannot definitively resolve the reason for this tissue-specific enrichment without deeper understanding of tissue-specific epitranscriptomic regulation, it is possible that colorectal tissues harbor biological features that increase the likelihood of interaction between germline variation and m^6^A modification. In addition, although variant burden alone does not fully explain the observed enrichment, differences in the number and genomic distribution of GWAS-identified SNPs across cancer types (Fig. 2B and C) may have contributed in part to detection sensitivity or statistical power. For example, although colon cancer harbored fewer associated SNPs than breast or prostate cancers, it showed significant enrichment, whereas other low-SNP cancers such as oral or renal did not. This suggests that variant burden may interact with tissue-specific regulatory environments to shape the observed enrichment pattern. Consistent with this interpretation, Liu et al. (2020) reported that m^6^A-related SNPs are strongly associated with colorectal cancer based on independent Disease Ontology enrichment analysis, reinforcing the biological plausibility of our findings^53^.

To explore potential functional consequences of m^6^A-associated SNPs, we focused on cancer-associated variants that overlapped with hypermethylated m^6^A peaks and were annotated as expression or splicing quantitative trait loci (eQTLs or sQTLs). These regions exhibited the strongest enrichment of disease-linked variants and showed frequent associations with changes in transcript abundance. In contrast, SNPs located in regions with decreased m^6^A methylation showed no comparable enrichment and were excluded from downstream analyses. Among the QTL-linked transcripts identified, *MCM3AP-AS1* and *RIBC2* were consistently upregulated in colon cancer.

The observed upregulation of *MCM3AP-AS1* and *RIBC2* was supported by both experimental and patient-derived data. In DLD1 and CCD841 cell lines, ALKBH5 depletion increased the expression of these transcripts, indicating that they are responsive to elevated m^6^A levels. Consistent expression changes were observed in an independent cohort of colon cancer patients (PRJNA742008), reinforcing the reproducibility of our findings across both experimental and clinical contexts. These results support the functional relevance of m^6^A in regulating these genes, in agreement with predictions from our integrative m^6^A-QTL analysis.

*MCM3AP-AS1* is a long noncoding RNA that has been implicated in the proliferation and invasion of colon cancer cells, potentially *via* Wnt/β-catenin^54^. *RIBC2*, although not previously characterized as a cancer-associated gene, also exhibited consistently elevated expression in colon cancer. While its role in oncogenesis remains to be experimentally validated, its regulation by m^6^A-sensitive loci highlights it as a gene of interest for future investigation.

We also observed that m^6^A-associated SNPs are linked to alternative splicing events in a manner that appears transcript- and context-dependent. In our discovery dataset, *FAM118A* and *TRIM4* exhibited A3SS usage in tumors. In an independent patient cohort, however, splicing patterns differed. In particular, *FAM118A* showed retained introns, while *TRIM4* exhibited exon skipping, indicating transcript-specific differences in splicing outcomes across datasets. These results suggest that m^6^A-SNP interactions may modulate splicing in variable ways depending on cellular context, RNA structure, or the availability of splicing regulators. Given the frequent positioning of m^6^A peaks within exons and promoter-proximal regions, these modifications may facilitate the recruitment of splicing-regulatory RNA-binding proteins, thereby influencing isoform selection in tumors.

Our findings extend current models of how noncoding genetic variants contribute to cancer biology. While many GWAS-identified SNPs lie outside protein-coding regions, their mechanistic interpretation has remained a major challenge. The present study provides evidence that m^6^A methylation may act as a regulatory intermediary, linking static DNA variation to dynamic RNA-level outcomes in tumor transcriptomes. Although tumor and normal tissues are nearly identical at the DNA sequence level, SNPs may shape m^6^A profiles that influence RNA stability, splicing, and abundance.

We define a class of functional variants, which we refer to as m^6^A-QTLs, that operate through epitranscriptomic mechanisms rather than classical transcriptional regulation. These variants reveal a genetically encoded layer of post-transcriptional control that is dynamic and cell-type specific. Although our findings were most prominent in colon cancer, this framework may also inform studies in other tumor types or molecular subgroups where m^6^A dysregulation and regulatory variation co-occur. Future work should further characterize m^6^A-QTLs across diverse biological contexts and investigate whether targeting m^6^A enzymes can mitigate the phenotypic effects of pathogenic variants. As small molecule inhibitors of m^6^A regulators move toward clinical development, our results underscore the importance of considering inherited variation in RNA regulatory mechanisms when designing targeted cancer therapies.

## Materials and methods

### Preprocessing of RNA-seq and m^6^A-seq data

Gene expression profiles (RNA-seq and m^6^A-seq) from tumor tissues and matched normal tissues for nine cancer types (including colorectal, breast, lung, ovarian, renal cell, and salivary gland cancers) were retrieved from the SRA (https://www.ncbi.nlm.nih.gov/sra) database as Fastq format. Detailed dataset information, including accession numbers and sample sizes is summarized in Supplementary Fig. S1A^32^. Fastq files underwent preprocessing with Trim-Galore (v.0.6.6; https://github.com/FelixKrueger/TrimGalore*)* to remove adapter sequence and low-quality reads, ensuring data quality for subsequent analyses. For RNA-seq data, processed reads were aligned to the human reference genome (GRCh38) with the STAR aligner (v.2.7.10a) using the GTF file from GENCODE (GRCh38.gencode.v43.basic.annotation.gtf) with default parameters^33^. Read counts of each gene was calculated using HTSeq package (v.2.0.2)^34^.

### Identification of differential m^6^A peaks (DMPs)

For m^6^A-seq data, peak calling was conducted using R package exomePeak (v.2.16.0)^37^, followed by annotation of peaks according to genomic features (UTR, exon, intron, promoter, and intergenic regions) using custom R scripts and the GENCODE hg38 annotation. Differential m^6^A peaks (DMPs) were identified using exomePeak with the following criteria: *P* < 0.05.

### Identification of differential expressed genes (DEGs)

For RNA-seq data, differentially expressed genes (DEGs) between tumor and normal tissues were identified using the DESeq2 R package (v.1.42.1)^35^. Genes with adjusted *P* value < 0.05 and absolute log2 fold-change > 0.58 were considered as DEG. Heatmaps were generated to visualize DEGs using the R packages ggplot2 (v.3.5.1) and pheatmap (v.1.0.12), respectively^55,56^.

### Identification of differentially spliced genes (DSGs)

To identify differentially spliced genes (DSGs) that contains alternative splicing events in tumor samples, RNA-seq reads were first aligned to the human reference genome (GENCODE hg38) using STAR (v.2.7.10a) with the following options (--twopassMode Basic --alignSJDBoverhangMin 1 --alignSJoverhangMin 8 --alignEndsType EndToEnd --outSAMattributes NH HI AS NM MD XS --outSAMstrandField intronMotif), which ensured high-confidence spliced alignment and preservation of strand-specific attributes required for downstream splicing analysis. The aligned RNA-seq data were analyzed using rMATS-turbo (v.4.1.2)^36^. Alternative splicing events including skipped exons (SE), retained introns (RI), alternative 5’ splice sites (A5SS), alternative 3’ splice sites (A3SS), and mutually exclusive exons (MXE) were quantified. Events with FDR < 0.05 were considered as statistically significant differential splicing events in tumors compared to normal tissues.

### Variant set enrichment analysis (VSEA)

Cancer-associated SNPs were obtained from the GWAS catalog^45^. Lead SNPs and their LD-associated SNPs (r^2^ ≥ 0.8) were extracted to establish a comprehensive set of cancer-specific genetic variants using R package haploR (v4.0.7) bioinformatics package^57^. To assess the functional significance of SNP enrichment within DMPs, Variant Set Enrichment Analysis (VSEA) was performed using the method previously described by Ahmed et al. (2017)^44^. Briefly, VSEA calculates enrichment scores by comparing the observed frequency of cancer-associated SNPs in DMPs to the estimated frequency of cancer-specific SNPs in the same number of randomly selected genomic regions. Statistical significance was determined by generation of null distributions based on 1,000 permutations and random selection of genomic regions each time, with *P* < 0.05 considered significant enrichment.

### eQTL and sQTL analysis

Expression quantitative trait loci (eQTL) and splicing quantitative trait loci (sQTL) analyses were conducted using snpXplorer^49^, an integrative database containing functional annotations of SNPs. Candidate cancer-associated SNPs overlapping with differential m^6^A peaks were queried against the snpXplorer database to narrow down SNPs that are potentially associated with the gene expression or alternative splicing events. Statistical significance of eQTL and sQTL associations was defined as *P* < 0.05.

### Functional validation via ALKBH5 knockdown

To validate the functional role of m^6^A methylation, ALKBH5 knockdown was performed using siRNA transfection (target-specific siRNA purchased from Genolution) in colon cancer (DLD1) and normal colon epithelial (CCD841) cell lines. Transfections were performed using Lipofectamine RNAiMAX reagent (Invitrogen) according to the manufacturer’s protocol. The following ALKBH5 siRNA sequence was used in this study: 5’-r(GCGCCGUCAUCAACGACUA)d(TT)-3’. Gene expression changes of selected targets were quantified *via* quantitative real-time PCR (qRT-PCR). Gene-specific oligonucleotides used in the study are as follows: 5’- TGGCAAATTCCATGGCACC-3’ (sense) and 5’- AGAGATGATGACCCTTTTG-3’ (antisense) for *GAPDH* mRNA; 5’- CAGAGAATGGTCTTTGCAGCAGC-3’ (sense) and 5’- TTCTGGAGGTGCTTGGCTGTCT-3’ (antisense) for *RIBC2* mRNA; and 5’- CTGGAAGCAGAAAGAGGCTGG-3’ (sense) and 5’- ACTGGAAGAGGAGCACAGAGTG-3’ (antisense) for *MCM3AP-AS1* lncRNA. All experiments were performed in triplicate.

### Statistical analysis

All statistical analyses were conducted using R software (v.4.2.1)^56^. Data are expressed as mean ± standard deviation. Comparisons between two groups were performed using Student’s t-tests. Spearman’s correlation analysis was performed using R packages ggpubr (v.0.6.0) and stats (base package)^58^. *P* < 0.05 was considered statistically significant.

## Supplementary Information

Below is the link to the electronic supplementary material.

Supplementary Material 1

Supplementary Material 2

Supplementary Material 3

Supplementary Material 4

## Data Availability

The RNA-seq and m^6^A-seq datasets analyzed in this study are publicly available from the NCBI Sequence Read Archive (SRA) under accession number PRJNA488293, PRJNA1011293, PRJNA659478, PRJNA679771, PRJNA901504, PRJNA814496, PRJNA719065, PRJNA1092401, PRJNA1039844, PRJNA786917, and PRJNA742008. Additional processed data supporting the findings of this study are available from the corresponding authors upon reasonable request.

## Abbreviations

A3SS: Alternative 3′ splice site
A5SS: Alternative 5′ splice site
CDS: Coding sequence
DEG/DEGs: Differentially expressed gene(s)
DMP/DMPs: Differential m^6^A peak(s)
DRACH: D = A/G/U, R = A/G, H = A/C/U
eQTL: Expression quantitative trait loci
FPKM: Fragments per kilobase of transcript per million mapped reads
GWAS: Genome-wide association study
LD: Linkage disequilibrium
lncRNA: Long non-coding RNA
m^6^A: *N6*-methyladenosine
m^6^A-seq: m^6^A RNA immunoprecipitation sequencing
MXE: Mutually exclusive exon
RI: Retained intron
RNA-seq: RNA sequencing
SE: Skipped exon
SNP: Single nucleotide polymorphism
sQTL: Splicing quantitative trait loci
SRA: Sequence read archive
TCGA: The cancer genome atlas
TTS: Transcription termination site
UTR: Untranslated region
VSEA: Variant Set Enrichment Analysis
