# Supplementary material for: Genetic variants reshape the m6A epitranscriptome and drive transcriptomic reprogramming in colorectal cancer
Source: Sci Rep. 2025 Nov 11;15:39384. doi: 10.1038/s41598-025-23397-x (PMC12606336; doi:10.1038/s41598-025-23397-x)
Supplement: Supplementary file 1 — Supplementary Material 1 [file 41598_2025_23397_MOESM1_ESM.pdf]

Genetic Variants Reshape the m^{6}A Epitranscriptome and Drive Transcriptomic Reprogramming in Colorectal Cancer

Seung Hun Han[1], Seongmin Jang[1], Yeongwon Kim[1], Kun Tan[2], Miles F. Wilkinson[2,3]*, Hyobin Jeong[4,*], and Junho Choe[1,5,6,7*]

[1]Department of Life Science, College of Natural Sciences, Hanyang University, Seoul 04763, Republic of Korea

[2]Department of Obstetrics, Gynecology, and Reproductive Sciences, School of Medicine, University of California San Diego, La Jolla, San Deigo, CA 92093, USA

[3]Institute of Genomic Medicine, University of California San Diego, La Jolla, San Diego, CA 92093, USA

[4]Department of Systems Biology, College of Life Science and Biotechnology, Yonsei University, Seoul, Republic of Korea

[5]Hanyang Institute of Bioscience and Biotechnology, Hanyang University, Seoul 04763, Republic of Korea

[6]Research Institute for Natural Sciences, Hanyang University, Seoul 04763, Republic of Korea

[7]Research Institute for Convergence of Basic Sciences, Hanyang University, Seoul 04763, Republic of Korea

* To whom correspondence should be addressed. Junho Choe (jcho2711@hanyang.ac.kr)

Correspondence may also be addressed to Miles F. Wilkinson (mfwilkinson@health.ucsd.edu); Hyobin Jeong (hyobinjeong@yonsei.ac.kr)

A

Type	Control	Patients	Study Accession
Ovarian Cancer	7	6	PRJNA488293
Cervical Cancer	1	3	PRJNA1011293
Oral Cancer	3	3	PRJNA659478
Salivary Cancer	5	5	PRJNA679771
Breast Cancer	2	2	PRJNA901504
Lung Cancer	3	3	PRJNA814496
Renal Cell Carcinoma	3	2	PRJNA719065
Prostate Cancer	3	3	PRJNA1092401
Colorectal Cancer	8	8	PRJNA1039844, PRJNA786917

C

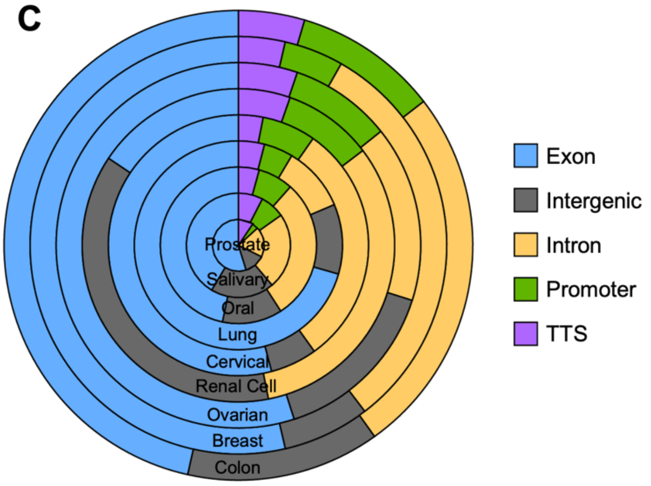

B

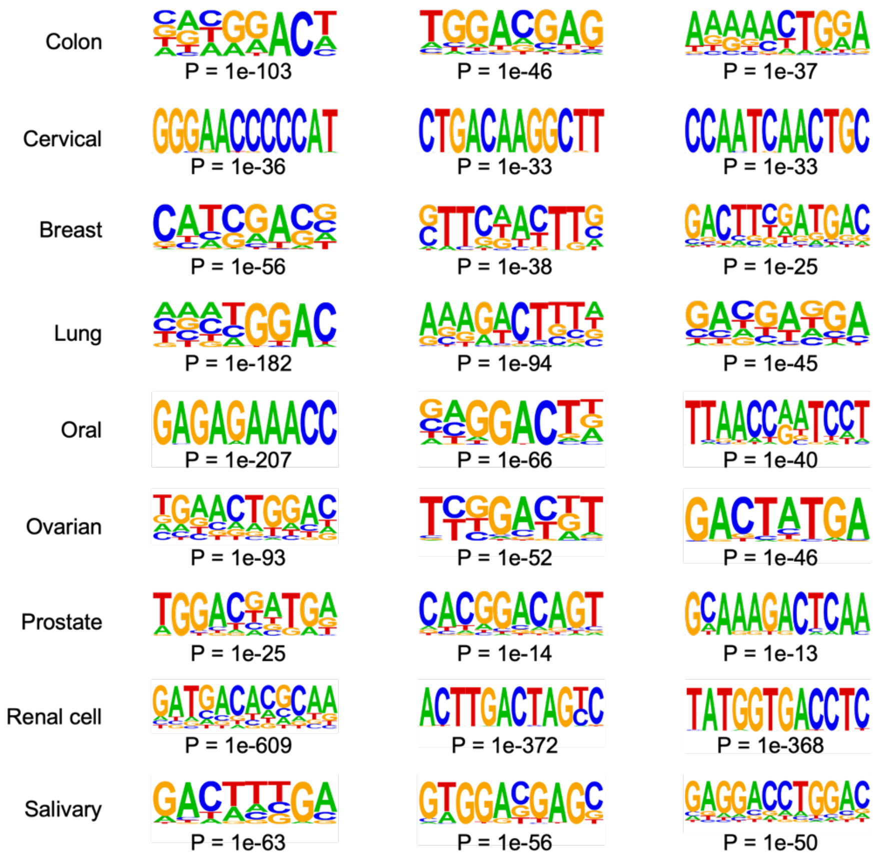

Supplementary Figure S1. Collection and quality assessment of m^{6}A-seq datasets in nine cancer types and normal tissues through the m^{6}A motif analysis. (A) The table shows cancer types, number of control and patient samples, and corresponding study accession numbers of publicly available transcriptome and epitranscriptome data sets used in this study. **(B)** Representative m^{6}A-enriched sequence motifs identified from m^{6}A peak regions

using HOMER (v.3.3) across each cancer type. **(C)** Genomic distribution of detected m^6A peaks using exomePeak across each cancer type.

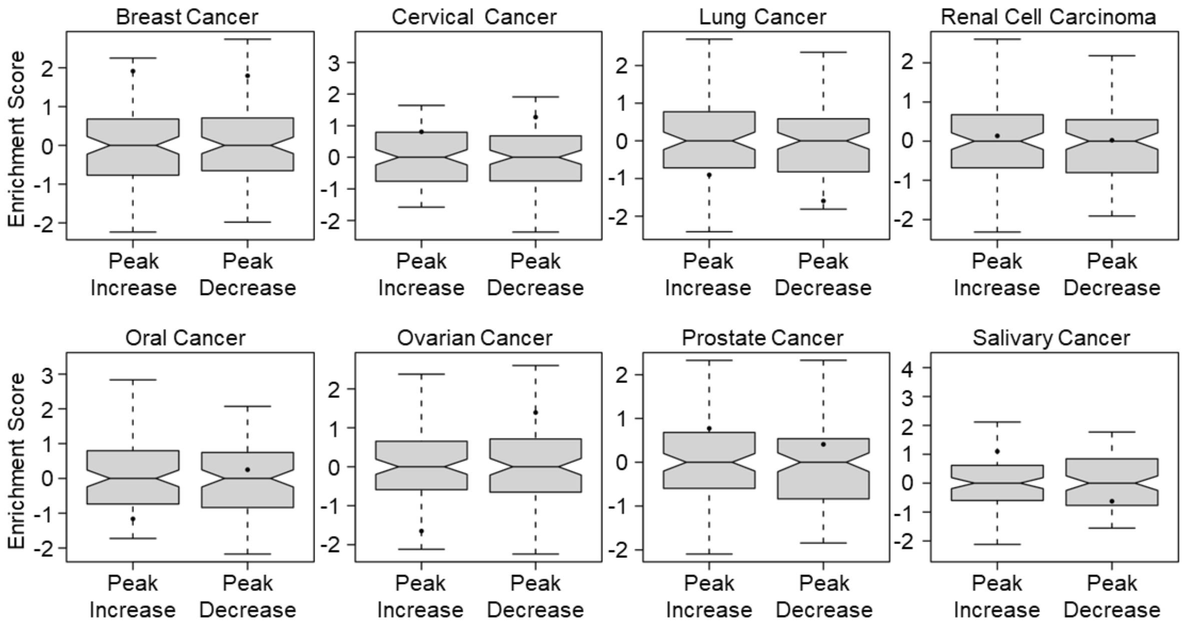

Supplementary Figure S2. VSEA to test the enrichment of cancer-associated SNPs within the increased or decreased m^{6}A peaks in multiple cancer type. VSEA results were shown for breast, cervical, lung, renal cell, oral, ovarian, prostate, and salivary gland cancer. No significant enrichment of cancer-associated SNPs was observed in any cancer type (adjusted *P* < 0.05; adjusted after Benjamini-Hochberg multiple corrections, based on permutation test, n = 1,000).

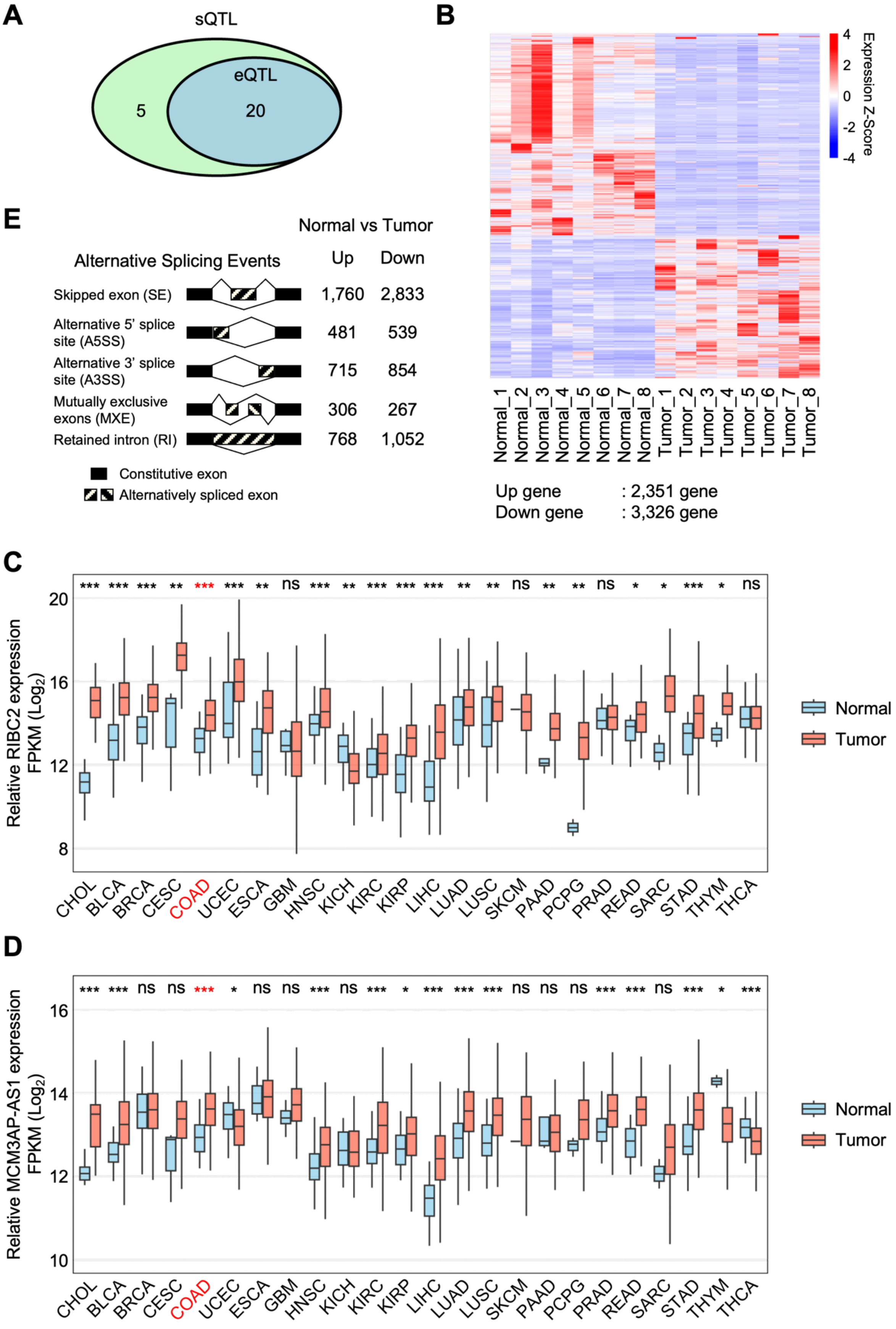
A
sQTL
eQTL
5
20
B
4
2
0
-2
-4
Expression Z-Score
Normal_1
Normal_2
Normal_3
Normal_4
Normal_5
Normal_6
Normal_7
Normal_8
Tumor_1
Tumor_2
Tumor_3
Tumor_4
Tumor_5
Tumor_6
Tumor_7
Tumor_8
Up gene : 2,351 gene
Down gene : 3,326 gene
E
Normal vs Tumor
Alternative Splicing Events
Up
Down
Skipped exon (SE) 1,760 2,833
Alternative 5' splice site (A5SS) 481 539
Alternative 3' splice site (A3SS) 715 854
Mutually exclusive exons (MXE) 306 267
Retained intron (RI) 768 1,052
Constitutive exon
Alternatively spliced exon
C
Relative RIBC2 expression FPKM (Log2)
*** *** *** ** *** *** ** ns *** ** *** *** *** ** ** ns ** ** ns * * *** * ns
20
16
12
8
CHOL
BLCA
BRCA
CESC
COAD
UCEC
ESCA
GBM
HNSC
KICH
KIRC
KIRP
LIHC
LUAD
LUSC
SKCM
PAAD
PCPG
PRAD
READ
SARC
STAD
THYM
THCA
Normal
Tumor
D
Relative MCM3AP-AS1 expression FPKM (Log2)
*** *** ns ns *** * ns ns *** ns *** * *** *** *** ns ns ns *** *** ns *** * ***
16
14
12
10
CHOL
BLCA
BRCA
CESC
COAD
UCEC
ESCA
GBM
HNSC
KICH
KIRC
KIRP
LIHC
LUAD
LUSC
SKCM
PAAD
PCPG
PRAD
READ
SARC
STAD
THYM
THCA
Normal
Tumor

Supplementary Figure S3. Transcriptomic and splicing alterations in colon cancer. (A) Venn diagram showing the overlap between m^6A-associated SNPs in colon cancer annotated as sQTL and eQTL. **(B)** Heatmap of differentially expressed genes between colon tumor and normal tissues, based on DESeq2 analysis of HTSeq counts. Genes with adjusted $P < 0.05$ and $|\log_2 FC| > 0.58$ were regarded as differentially expressed genes (DEGs). The number of up and down DEGs were shown below. **(C)** TCGA-based expression analysis of *RIBC2* across various cancers, with elevated expression observed in colon adenocarcinoma (COAD). **(D)** TCGA-based expression analysis of *MCM3AP-AS1*, also showing upregulation in COAD compared to normal tissues. The significance of differential expressions between tumor and normal tissues were determined by two-sided Wilcoxon rank-sum test. **(E)** Global alternative splicing changes identified by rMATS-turbo, categorized by splicing event type. The asterisks indicate statistical significance: * $P < 0.05$, ** $P < 0.01$, *** $P < 0.001$.

Supplementary Table S1. Statistical significance of VSEA results after multiple testing correction. AVS: refers to predefined collection of genetic variants. The AVSs included in this analysis were those found to overlap with m^6A peaks. (Table accompanying the submission as spreadsheet.)

Supplementary Table S2. List of colon cancer-associated SNPs, overlapping with colon cancer m^6A peaks. NA: indicates values that are either not available due to lack of data or not applicable for the specific SNP or gene. (Table accompanying the submission as spreadsheet.)

Supplementary Table S3. List of colon cancer-associated m^6A-SNPs within the *RIBC2* and *MCM3AP-AS1* locus, derived from integrative search of m^6A peaks and HaploReg database. (Table accompanying the submission as spreadsheet.)
